# Application of single-port procedure and ERAS management in the laparoscopic myomectomy

**DOI:** 10.1186/s12905-023-02550-6

**Published:** 2023-08-01

**Authors:** Jing Wang, Xiaomin Xu, Jingui Xu

**Affiliations:** Department of Gynecology, The Quzhou Affiliated Hospital of Wenzhou Medical University, Quzhou People’s Hospital, No.100 Minjiang Avenue, Kecheng District, Quzhou City, 324000 Zhejiang Province China

**Keywords:** Enhanced recovery after surgery, Laparoscopy, Myomectomy

## Abstract

**Objective:**

Advances in surgical techniques and perioperative management are the two major contributing factors to improved surgical outcomes. The purpose of the current study was to compare the efficacy of single-port surgery and perioperative enhanced recovery after surgery (ERAS) management in laparoscopic myomectomy.

**Methods:**

The present study included 120 patients undergoing laparoscopic myomectomy in the Gynecological Ward of Quzhou Affiliated Hospital of Wenzhou Medical University. According to the traditional perioperative management mode and ERAS management, multi-port and single-port procedures, all patients were assigned to the Conventional-SPLS (Single-Port Laparoscopic Surgery with conventional perioperative care) group (n = 34), Conventional-Multi (multi-port laparoscopic surgery with conventional perioperative care) group (n = 47), and ERAS (multi-port laparoscopic surgery with ERAS perioperative care) group (n = 39). The surgical outcomes of the three groups were compared operation time, intraoperative blood loss, variations in postoperative hemoglobin, postoperative walking time, postoperative flatus expelling time, postoperative hospital stay, and visual analog scale (VAS) scores at 6 and 12 h following surgery.

**Results:**

The ERAS group recovered the quickest in terms of postoperative walking time and flatus expelling duration. The ERAS group also recovered the shortest postoperative hospital stay (3.85 ± 1.14 days), which differed significantly from that in the Conventional-Multi group, but not significantly from that in the Conventional-SPLS group. In terms of VAS scores at 6 and 12 h after surgery, the ERAS group had the lowest pain intensity, which differed significantly from that of the other two groups. The effect of surgical procedures or postoperative care on hospital stay was assessed using multiple regression analysis. The results demonstrated that ERAS was an important independent contributor to reducing postoperative hospital stay (β = 0.270, p = 0.002), while single-port surgery did not affect this index (β = 0.107, p = 0.278).

**Conclusion:**

In laparoscopic myomectomy, perioperative ERAS management could control postoperative pain and shorten hospital stay. Single-port surgery could speed up the recovery of gastrointestinal function and postoperative walking time, but it did not affect postoperative pain management or the length of hospital stay. Thus, the most effective approach to improving postoperative outcomes in laparoscopic myomectomy was the application of perioperative ERAS management.

## Introduction

The myomectomy can preserve fertility and maintain the anatomical integrity of the pelvic floor. Patients are increasingly selecting laparoscopic myomectomy because of the rapid advancement of minimally invasive procedures. However, using a fibroid morcellator and other issues limit the application of this procedure. The laparoscopic electric fibroid morcellator has been widely used in laparoscopic myomectomy since the U.S. Food and Drug Administration (FDA) approved its clinical use in 1995. As the widespread application, related problems have attracted increasing attention. The high-speed rotating blades of the fibroid morcellator may damage surrounding organs, and the incidence is 0.007-0.02% [1]. It may also lead to the dissemination of lesions, such as parasitic leiomyomata, iatrogenic endometriosis, and cancer progression [2]. Qin Chen et al. retrospectively reviewed the data of 4478 patients undergoing laparoscopic myomectomy, and the incidence of uterine sarcomas was 0.54%. Uterine sarcoma incidence in people aged 50 to 60 years was as high as 10/375 (2.6%), and using a fibroid morcellator increased the risk of malignant tumors spreading to the abdominopelvic cavity [3]. Thus, the FDA stated the application of fibroid morcellator and warnings in 2014, limiting the application of laparoscopic myomectomy.

In recent years, transumbilical single-port laparoscopic surgery has undergone rapid development. This procedure makes an incision in the umbilical region that is 2.5-3.0 cm long. With the aid of an “apple-peeling” technique, the fibroids are taken out, placed in a retrieval bag, and then taken out of the incision. This procedure avoids problems associated with the use of a fibroid morcellator and the potential risk of lesion dissemination, and it is also more aesthetically pleasing and safer [4]. However, it necessitates more advanced laparoscopic techniques for surgeons, a longer duration of surgery, and a learning curve [5, 6].

Historically, the classic motto of postoperative management was “wait and see”. There was little data on perioperative care, such as intestinal preparation, dietary management, pain control, early mobilization, etc. In recent years, there has been a paradigm shift towards a more positive attitude. ERAS was first described by Danish surgeon Kehlet in 1997 [7], and using the principles of evidence-based medicine, it made several perioperative adjustments to speed the recovery of various tissues and organs after surgery [8].The goal of ERAS is to reduce the physiological pressure of surgery and optimize patient recovery. Before surgery, this is achieved by optimizing chronic diseases and nutrition,counseling and education,no mechanical bowel preparation,oral carbohydrate loading. During the surgical process, the goal is to minimize pain and gastrointestinal dysfunction through preferred minimally invasive methods, anesthetic management,temperature regulation, and goal oriented fluid management. After surgery, the goals include good nutrition, early mobilization, and early removal of packaging, drainage tubes, and catheters. The ultimate goal of this method is to allow patients to resume normal activities faster while saving costs, but without affecting patient satisfaction or quality of care [9].

Both strategies can enhance the results of a laparoscopic myomectomy, but only one call for the cooperation of specialists from various fields,which means that the institution must be large enough to establish a multidisciplinary team,while the other relies on the skill of the surgeons. Multi-port laparoscopic surgery and traditional postoperative care are currently the two main surgical options in China for uterine fibroids. To further improve surgical outcomes, investigations are warranted to compare their effectiveness. In this setting, the present study aimed to compare the effectiveness of ERAS (enhanced management approach) and single-port laparoscopic surgery (enhanced technique approach) in improving postoperative outcomes.

## Materials and methods

### Patients

This was a retrospective single-center study involving 120 patients undergoing laparoscopic myomectomy in the Gynecological Ward of Quzhou Affiliated Hospital of Wenzhou Medical University. From January 2020 to December 2021, 34 patients in the Conventional-SPLS (Single-Port Laparoscopic Surgery with conventional perioperative care) group and 47 patients in the Conventional-Multi group (multi-port laparoscopic surgery with conventional perioperative care) were enrolled. From January 2022, the Gynecological Ward of this hospital adopted the EARS mode, so 39 in the ERAS group (multi-port laparoscopic surgery with ERAS perioperative care) were enrolled from January 2022 to December 2022.

Inclusion criteria were as follows: (1) symptomatic (such as abnormal uterine bleeding, compression symptoms, infertility, etc.) uterine fibroids confirmed by transvaginal color Doppler ultrasonography or pelvic magnetic resonance imaging (MRI); (2) The number of fibroids was less than 5, and the maximum diameter of fibroid was less than or equal to 10 cm; (3) The preoperative hemoglobin level was within the normal range. Exclusion criteria were as follows: (1) other surgeries except for myomectomy; (2) other chronic diseases, such as diabetes, hypertension, and so forth. The Institutional Review Board of this hospital gave its approval to the current study. Each procedure was carried out by surgeons with prior experience in laparoscopic single-port and multi-port procedures.

### Surgical procedure

After the successful induction of general anesthesia, the patient was laid out in the supine position while standard cleaning and dressing procedures were carried out.

The transumbilical single-port laparoscopic surgery group employed a custom “glove approach,“ making a vertical longitudinal incision through the umbilical region measuring approximately 2.5-3.0 cm in length. Next, a protective sleeve for the abdominal wall incision was then inserted after cutting through a full-thickness abdominal wall to reveal the abdominal cavity. After wrapping its outer ring in sterile latex gloves, three glove fingertips were cut, and one 1.0 cm and two 0.5 cm trocars were inserted before being secured with silk suture. The pressure was kept at 1.6 kPa while the CO_2_ pneumoperitoneum formed (12 mmHg). During the procedure, a 30° laparoscopic lens was used, as well as a 30° Trendelenburg position. Intraoperatively, the size and location of uterine fibroids were investigated, and pituitrin 6 U diluted with 10 ml of normal saline was injected into the uterine body; then, an absorbable suture (ETHICON, SXPP1A405) was used to layer-suture the tumor cavity after making a longitudinal incision along the surface of the fibroids with a monopolar electric hook that was the same diameter as the fibroids. Following the removal of the fibroids, the tissues were collected and taken out of the umbilical incision using the retrieval bag. In the umbilical incision, 2 − 0 absorbable sutures were used to close the peritoneal, fascial, and skin layers.

In multi-port laparoscopic surgery group, one 10-mm trocar was inserted at the umbilicus,one 5-mm and one 12-mm trocars were inserted at the left lower abdomen. Another 5-mm trocar was inserted in the right lower quadrant of the abdomen depending on the surgeon’s preference.Myomas were extracted through the 12-mm trocar site with a 12-mm electromechanical power morcellator without a bag. All other procedures were similar to the single-port surgery, except for port placement and extraction.

### Perioperative management

#### Preoperative education

The ERAS group focused on communication with patients, adopting a “one-to-one” mode, as well as promotional materials, pictures, videos, and other methods. In-depth introductions were given to the anticipated objectives of ERAS, admission preparation, perioperative procedures (including surgery and anesthesia), steps requiring patients’ cooperation, postoperative rehabilitation, and discharge criteria. In the traditional groups, patients were only informed of the necessity for surgery, the risks involved, and how to reduce them.

#### Preoperative bowel preparation, dietary control and oral carbohydrates

In the ERAS group, bowel preparation was not a common practice. The day before surgery, a normal diet was begun, and dairy products and starchy solid food were avoided for six hours, while bland liquid food was avoided for two hours. Two hours before surgery, the patient drank an appropriate amount of a maltodextrin-containing carbohydrate beverage. After postoperative waking up, a small amount of warm water could be drunk, and liquid or semi-liquid food could be taken 6 h after surgery; moreover, intermittent chewing gum was encouraged. Traditional groups practiced routine bowel preparation and bowel cleansing preparation. In addition, 12 h were spent without food or water before surgery; following flatus evacuation, food was consumed, and the diet was gradually changed.

#### Anesthetic management

The ERAS group employed combined intravenous-inhalation anesthesia, administered anesthetics with brief half-lives, and avoided the use of opioid analgesics. The traditional groups received traditional general anesthesia.

#### Body temperature management

In the ERAS group, the exposure was reduced while maintaining a constant room temperature of 25 °C during surgery; the patient’s temperature balance was maintained by various methods such as fan heaters, insulation blankets, warming infusion devices, and peritoneal washes for heating to ensure that the temperature was above 36 °C when leaving the operating room. The traditional groups did not receive body-temperature management.

#### Catheter indwelling time

In the ERAS group, the urinary catheter was removed 6 h after surgery, whereas in the traditional groups, it was removed 24 or 48 h later.

#### Preemptive analgesia and multi-modal, regular analgesia

The multi-modal sufficient analgesia, primarily based on NSAIDS, was carried out in the ERAS group. Before surgery, a 30-minute pain intervention was given, and based on the level of pain, the appropriate treatment was administered. Corresponding treatments were performed in the traditional groups when pain occurred.

#### Postoperative early ambulation

The ERAS group underwent postoperative head elevation (20–30°), passive and active lower limb movements, movement encouragement on the day of surgery, and off-bed movement requirements on the first postoperative day. Early ambulation was encouraged but not strictly required in the traditional groups.

### Observation index

Preoperative indices: Patients’ age, body mass index, parity, and several pelvic surgeries were recorded.

Intraoperative indices: The number of fibroids, the largest fibroid diameter, the length of the operation (from the beginning to the end of the operation), and the intraoperative blood loss (the volumetric method was used to determine the difference between the volume of fluid in the aspirator and the volume of flushing liquid) were all noted.

Postoperative indices: Variations in postoperative hemoglobin, postoperative walking time, postoperative flatus expelling time, postoperative hospital stay, and visual analog scale (VAS) scores at 6 and 12 h after the surgery was recorded. The VAS score, which ranges from 0 (no pain) to 10 (extremely severe pain), was used to measure the intensity of the pain.

### Statistical analysis

Using the software SPSS 26.0, data processing and statistical analyses were carried out; the measurement data were presented as xˉ ± s; the comparison between the three groups was done using a one-way analysis of variance. The impact of single-port laparoscopic surgery and perioperative ERAS management on the length of the patient’s stay in the hospital following surgery was assessed using multiple regression analysis. *P <* 0.05 was considered statistically significant, and *P <* 0.01 was considered highly statistically significant.

## Results

### Patient’s characteristics

A total of 120 patients were enrolled, including 34 in the Conventional-SPLS group, 47 in the Conventional-Multi group, and 39 in the ERAS group. Age, BMI, parity, and the number of prior pelvic surgeries did not statistically significantly differ among the three groups (Table 1).

**Table 1:** Clinical Characteristics of Patients in 3 Groups

Characteristics	Conventional-Multi (n = 47)	Conventional-SPLS (n = 34)	ERAS (n = 39)	*p*-value
Age	39.26 ± 5.41	38.15 ± 5.55	38.72 ± 5.72	0.674
BMI	22.57 ± 2.79	22.77 ± 2.97	23.08 ± 2.15	0.678
Parity	1.28 ± 0.68	1.41 ± 0.74	1.31 ± 0.73	0.692
No.of previous abdominopelvic surgery	0.43 ± 0.65	0.47 ± 0.62	0.62 ± 0.67	0.385

Data presented as mean ± SD.

BMI:body mass index;ERAS:Enhanced Recovery After Surgery;SPLS:Single-Port Laparoscopic Surgery

### Operation index

During the procedure, the number of fibroids in the Conventional-SPLS group was 1.53 ± 0.79, which was less than that in the other two groups (1.87 ± 1.14 in the Conventional-Multi group and 1.77 ± 1.10 in the ERAS group); the maximum fibroid diameter in the Conventional-SPLS group was 6.53 ± 1.01, which was also smaller (6.82 ± 1.38 in the Conventional-Multi group, and 6.82 ± 1.09 in the ERAS group), but there were no statistical differences. The duration of surgery in the Conventional-SPLS group was 79.85 ± 12.94 min, which was significantly longer than that in the Conventional-Multi group (70.26 ± 12.66 min), which had a statistical difference. Additionally, there were significant differences for pairwise comparisons in the postoperative walking time and postoperative flatus expelling time among the three groups, but not in blood loss or postoperative hemoglobin changes. Furthermore, the ERAS group recovered the fastest, with the shortest postoperative walking time and flatus expelling time. The shortest postoperative hospital stay in the ERAS group was 3.85 ± 1.14 days, which differed significantly from that in the Conventional-Multi group, but not significantly from that in the Conventional-SPLS group. The ERAS group had the least pain intensity on the VAS scale at 6- and 12 h following surgery, which was significantly different from the pain intensity of the other two groups (Table 2).

**Table 2:** Perioperative outcomes in 3 Groups

Characteristics	Conventional -Multi (n = 47)	Conventional-SPLS (n = 34)	ERAS (n = 39)	†*p*	‡*p*	§*p*	‖*p*
No. of myomas	1.87 ± 1.14	1.53 ± 0.79	1.77 ± 1.10	0.33	0.143	0.645	0.324
Size of myomas (cm)	6.82 ± 1.38	6.53 ± 1.01	6.82 ± 1.09	0.487	0.301	0.964	0.627
Operation time (min)	70.26 ± 12.66	79.85 ± 12.94	74.36 ± 14.65	0.008**	0.002**	0.160	0.083
Blood loss (ml)	119.47 ± 53.78	121.18 ± 81.46	113.21 ± 57.41	0.849	0.906	0.652	0.596
Hemoglobin change after operation(g/L)	14.15 ± 5.95	12.56 ± 6.18	12.00 ± 5.06	0.200	0.221	0.087	0.679
Postoperative walking time (d)	20.06 ± 3.84	17.68 ± 3.78	11.97 ± 4.02	0.000**	0.007**	0.000**	0.000**
Postoperative flatus expelling time (d)	22.04 ± 3.59	19.76 ± 4.21	15.10 ± 3.87	0.000**	0.010*	0.000**	0.000**
Postoperative hospital stay (d)	4.81 ± 0.82	4.35 ± 1.37	3.85 ± 1.14	0.000**	0.069	0.000**	0.052
Postoperative pain score (VAS)							
At 6 h	3.60 ± 0.85	3.47 ± 0.66	2.69 ± 1.00	0.000**	0.518	0.000**	0.000**
At 12 h	2.77 ± 0.73	2.65 ± 0.92	1.44 ± 0.55	0.000**	0.476	0.000**	0.000**

Data presented as mean ± SD.

ERAS:Enhanced Recovery After Surgery;SPLS:Single-Port Laparoscopic Surgery;VAS:Visual analogue scale

†*p*-value comparison in the 3 groups

‡*p*-value comparison between Conventional -Multi and Conventional-SPLS.

§*p*-value comparison between Conventional -Multi and ERAS.

‖*p*-value comparison betweenConventional-SPLS and ERAS.

*p < 0.05

**p < 0.01

Multiple regression analysis was used to assess the impact of surgical procedures or perioperative management on hospital stay. The results demonstrated that ERAS was an important independent contributor to reducing postoperative hospital stay (β = 0.270, p = 0.002). Another contributor to hospital stay was intraoperative blood loss (β = 0.369, p = 0.007). This index was unaffected by the type of surgery performed, whether it was single-port or multiple-port laparoscopic (β = 0.107, p = 0.278) (Table 3).

**Table 3:** Multiple regression analysis for evaluating significant factors on postoperative length of hospital stay

	Unstandardized Coefficients		Standardized Coefficients	t	Sig.
	B	Std. Error	Beta		
(Constant)	3.863	1.410		2.740	0.007
Age	0.025	0.019	0.116	1.281	0.203
BMI	− 0.051	0.036	− 0.116	-1.405	0.163
Parity	− 0.214	0.160	− 0.131	-1.338	0.184
No.of previous abdominopelvic surgery	− 0.065	0.164	− 0.036	− 0.397	0.692
No. of myomas	0.056	0.175	0.049	0.318	0.751
Size of myomas (cm)	− 0.029	0.109	− 0.029	− 0.266	0.791
Operation time (min)	− 0.016	0.013	− 0.188	-1.196	0.234
Blood loss (ml)	0.007	0.002	0.369	2.726	0.007
ERAS	0.669	0.209	0.270	3.193	0.002
SPLS	0.251	0.230	0.107	1.089	0.278

BMI:body mass index;ERAS:Enhanced Recovery After Surgery;SPLS:Single-Port Laparoscopic Surgery

## Discussion

Advances in surgical techniques and improvements in perioperative management are the two major contributing factors to improved surgical outcomes.

The development of minimally invasive concepts and aesthetic humanistic care in gynecological surgery has led to an increase in the use of single-port laparoscopic surgery. Transumbilical single-port laparoscopic myomectomy uses intrinsic scars to complete the procedure via the umbilicus. It can realize micro-scar surgical effects and is convenient to remove myomas, avoiding the problems caused by the morcellation of traditional multi-port laparoscopic surgery. However, the use of this technique in gynecology is still in the experimental stage due to the linear field of view, mutual interference of instruments, suturing technique, and challenging operation in single-port laparoscopic surgery. Studies by Su Mi Kim et al. [10]. have shown that single-port myomectomy with transumbilical myoma morcellation is feasible and safe, with outcomes comparable to those of three-port myomectomy. Studies by Lili Jiang et al [5]. suggested that compared with the traditional three-port laparoscopic group, the specimen removal time, postoperative ambulation time, first exhaust time after surgery, the length of hospital stay were all shorter, the satisfaction of abdominal wall scar were higher in the single-port laparoscopic group. The duration of surgery was longer in the single-port laparoscopic group significantly. There were no differences between the two groups’ pain scores on the day of surgery and the first day following surgery (*P* > 0.05) or the intraoperative blood loss, hemoglobin change, or postoperative hemoglobin changes. Dayong Lee et al. thinks [11] that single-port laparoscopic myomectomy was associated with more favorable cosmetic outcomes and patient satisfaction compared to conventional laparoscopic myomectomy. There were no differences in operative outcomes and complications between the two modalities. A meta-analysis [12] revealed that single-port laparoscopic myomectomy was superior to conventional laparoscopic myomectomy in terms of immediate postoperative pain relief while being equally safe and feasible. Single port laparoscopic myomectomy can have similar surgical outcomes to conventional laparoscopic myomectomy if performed according to the appropriate patient selection criteria, such as size and the number of myomas. The present findings demonstrated that the single-port laparoscopic surgery had longer surgery time (p = 0.002), shorter postoperative walking time, and flatus expelling time compared with the multi-port laparoscopic surgery, but there were no significant differences in VAS scores and hospital stay, which were consistent with the findings of Dayong Lee et al. [11].

More studies on the ERAS, another perioperative management strategy to improve surgical outcomes, have been published recently. The majority of surgical specialties worldwide have embraced this concept, which has been successfully implemented in a variety of surgical fields. In 2016, Nelson et al. issued guidelines for the application of ERAS in the perioperative period of gynecology and gynecological tumors, which for the first time comprehensively and systematically summarized the key points of the application of ERAS in gynecology [13, 14]. According to the characteristics of their respective disciplines, the various discipline branches of the Chinese Medical Association have gradually improved the expert consensus and guidelines in multiple disciplines. One of them, the *Consensus Guidelines for Enhanced Recovery After Gynecologic Surgery*, was proposed in 2019 [15]. Multiple systematic reviews and meta-analysis [8, 16]showed that ERAS pathways significantly reduced the length of stay in gynecological surgery and improved patient satisfaction. With a focus on the patient’s overall health and increasing specialist doctor’s awareness of the general practice, ERAS also highlighted the entire patient management process. Its primary goals were to preserve preoperative organ function, lessen severe stress reactions, and thoroughly restore the entire body using a variety of perioperative improvement techniques [9, 17]. Nonetheless, ERAS has been reluctantly adopted by medical institutions in actual practice because this approach requires surgeons to establish a multidisciplinary team, which means that the institution must be large enough to support such a team. The Gynecological Ward of this hospital used the ERAS management mode for surgical patients since 2022, so all patients in the ERAS group were enrolled after 2022. In the current study, the VAS score of the ERAS group was lower than that of the other two groups, and the difference was statistically significant. However, there was no significant statistical difference in VAS between Conventional-SPLS group and Conventional-Multi group.This finding was consistent with Buzzaccarini G et al [18]. It suggested that the sole surgical technique can be insufficient for determining the best option in terms of pain reduction.A large systematic review and meta-analysis also showed no significant difference between single-port laparoscopy and the traditional laparoscopic approach in terms of postoperative pain at 6 and 24 h related to adnexal surgery [19]. The ERAS group’s postoperative walking and flatus expelling times were also noticeably less than those of the other two groups. However, the ERAS group and the Conventional-Multi group had a significantly shorter postoperative hospital stay, but there was no significant difference compared with the Conventional-SPLS group.

In the present study, it suggested that the postoperative hospital stay was shorter in the ERAS group; compared with traditional multi-port laparoscopic surgery and traditional perioperative management, the ERAS group strengthened the perioperative management but did not result in improvement compared with the SPLS group, suggesting that the single-port procedure was still effective. Thus, it remains unclear regarding factors significantly improving postoperative outcomes. Multiple regression analysis was performed in this situation, and the results indicate that ERAS was a significant independent factor in reducing postoperative hospital stay (β = 0.270, p = 0.002). This finding was consistent with a systematic review and meta-analysis, which showed ERAS protocols can decrease length of stay, complications, and cost without increasing rates of readmission or mortality [20]. Another factor affecting postoperative hospital stay was intraoperative blood loss. Thus, during surgery, single-port or multi-port procedures must be accurate to reduce blood loss. Moreover, surgical techniques, regardless of single-port or multi-port laparoscopic surgery did not affect postoperative hospital stay (β = 0.107, p = 0.278). For some individuals, single-port laparoscopy may be suggested as an alternative to conventional multiport laparoscopy since it was considered as a feasible and reliable technique. It had lower postoperative pain scores and better scar satisfaction scores. A single incision around the umbilicus would not leave an unsightly scar, and the wound healing period would be shorter, minimizing the length of hospital stay [21]. A systematic review summarized the benefits and drawbacks in benign gynecological surgeries by single-port laparoscopy and conventional multiport laparoscopy, respectively. For myomectomy, there was no difference in the length of hospitalization, postoperative pain, the only significant difference was found for a higher conversion rate in the single-port group [6]. This indicated that we still need to be rigorous in single-port surgery.

Oue study suggests that perioperative ERAS management supports postoperative recovery more than the single-port surgical technique. But it’s critical to acknowledge the impact of the single-port procedure on early postoperative recovery (postoperative walking time and flatus expelling time), as well as the concept of a tumor-free body.

The present study has several limitations: the treatment time is different between the ERAS group and the other two groups, and it is a small retrospective study. Thus, it is impossible to avoid selection bias. This bias is lessened, though, by selecting test subjects who satisfy the same eligibility requirements. In patients undergoing ERAS, hospital discharge needs to meet definite criteria tolerance to a soft diet, ability to walk without assistance, no evidence of complications, and so forth, but in Conventional-SPLS or Conventional-Multi group, the discharge depends on the surgeon’s clinical assessment for each situation. In addition, these surgeries were performed by two surgical teams, which is also a limitation.Despite these limitations, the implications of the present findings are definite.

## Conclusion

In laparoscopic myomectomy, perioperative ERAS management can control postoperative pain and shorten hospital stays. Single-port surgery can speed up the recovery of gastrointestinal function and postoperative walking time, however, it does not affect postoperative pain management or hospital stay length. Thus, the most effective approach to improving postoperative outcomes in laparoscopic myomectomy is the application of perioperative ERAS management.

## Data Availability

The data used to support the findings of this study are available from the corresponding author upon request.

## List of abbreviations

ERAS: enhanced recovery after surgery
SPLS: Single-Port Laparoscopic Surgery
VAS: visual analog scale
